# Comparative bactericidal activity of four macrolides alone and combined with rifampicin or doxycycline against *Rhodococcus equi* at concentrations achievable in foals

**DOI:** 10.3389/fphar.2024.1458496

**Published:** 2024-11-18

**Authors:** Anne-Sophie Huguet, Ophélie Gourbeyre, Agathe Bernand, Charline Philibert, Alain Bousquet-Melou, Elodie A. Lallemand, Aude A. Ferran

**Affiliations:** INTHERES, INRAE, ENVT, University of Toulouse, Toulouse, France

**Keywords:** foal, drug combination, synergy, lung, macrophage, lung cells

## Abstract

**Introduction:**

*Rhodococcus equi* causes life-threatening respiratory disease in foals. The standard treatment typically involves a combination of rifampicin and a macrolide antibiotic. Although previous studies have demonstrated the *in vitro* activity of these antibiotics against *Rhodococcus equi*, the tested concentrations often do not reflect those achievable in foals.

**Material and Methods:**

Therefore, this study was performed to evaluate the *in vitro* bactericidal activity of rifampicin, doxycycline, and four macrolides (clarithromycin, azithromycin, gamithromycin and tulathromycin) individually and in combination, at concentrations observed at the target site of infection in foals. Additionally, we investigated the efficacy of these antibiotics at different pH levels to replicate the conditions in the pulmonary epithelial lining fluid and within macrophages, where *R. equi* can reside. We assessed the activity of antibiotics against a virulent strain of *R. equi* by determining the minimum inhibitory concentration (MIC) and performing checkerboard and time-kill curve assays with drugs both alone and in combination.

**Results:**

Time-kill curves with rifampicin or doxycycline demonstrated a reduction in *R. equi* counts by more than 3 log_10_ CFU/mL. Among the macrolides, tulathromycin was ineffective, while clarithromycin achieved bacterial elimination within 24 h under both extracellular and intracellular conditions. Gamithromycin and azithromycin exhibited bactericidal activity only in extracellular conditions, with no effect on the bacteria at pH 5.8. The checkerboard assay did not reveal any strong synergistic or antagonistic effects for rifampicin or doxycycline when combined with macrolides. In time-kill curves performed with maximal local concentrations achievable in foals, the combinations of rifampicin or doxycycline with macrolides did not increase the bacterial killing rate compared with the drugs alone, except for the combination of rifampicin with azithromycin, which showed slightly faster activity. However, the lower concentrations of doxycycline and clarithromycin that might be present 24 h after treatment in foals were effective in killing bacteria under intracellular conditions only when used in combination, and not when used alone.

**Conclusion:**

Our study suggests that clarithromycin can be used either alone or with doxycycline and that its use in combination with rifampicin should be reconsidered. Nevertheless, further studies are required to assess the clinical efficacy and potential side effects of doxycycline in foals.

## 1 Introduction

*Rhodococcus equi* (*R. equi*) is a Gram-positive, facultative intracellular bacterium commonly found in the environment and in the gastrointestinal tract of horses (Barton and Hughes, 1984). It is the causative agent of rhodococcosis, a life-threatening respiratory disease characterised by chronic granulomatous bronchopneumonia and lung abscesses in foals aged 3 weeks to 5 months (Giguère et al., 2012; Giguère et al., 2015; Sanz, 2023; Muscatello, 2012). Extrapulmonary manifestations can also occur, including vertebral osteomyelitis, septic pleuritis, mediastinal lymphadenopathy, uveitis and hypopyon (Reuss et al., 2009). This infection has a significant global economic impact on the equine industry due to the high costs associated with prolonged treatment and the absence of an effective vaccine. The pathogenicity of *R. equi* arises from its ability to survive and replicate intracellularly within mononuclear phagocytes (Hondalus and Mosser, 1994), a capability that is mediated by a plasmid carrying a gene encoding a highly immunogenic surface protein known as virulence-associated protein A (VapA) (Takai et al., 1991).

*In vitro*, various antimicrobial agents have shown activity against *R. equi* (Giguère et al., 2012; Giguère, 2017; Carlson et al., 2010; Prescott and Nicholson, 1984). However, many of these drugs are reported to be ineffective *in vivo*, likely because of poor cellular uptake leading to low intracellular concentrations (Giguère et al., 2015). Additionally, some antibiotics that could potentially be effective against *R. equi* may cause significant adverse effects in foals; for instance, gentamicin can lead to renal toxicity (Riviere et al., 1983), while enrofloxacin poses a risk of arthropathy (Vivrette et al., 2001). Historically, the recommended treatment for *R. equi* infections involved combining rifampicin with erythromycin to achieve a synergistic effect (Giguère et al., 2012; Prescott and Nicholson, 1984; Lakritz et al., 2000) and to prevent the rapid development of resistance associated with rifampicin monotherapy (Berghaus et al., 2013). Subsequently, erythromycin was largely replaced by azithromycin or clarithromycin, two macrolides with higher bioavailability following oral administration in foals, which may reduce adverse effects on the commensal flora (Giguére et al., 2004; Villarino and Martín‐Jiménez, 2013). More recently, two additional macrolides, gamithromycin (Hildebrand et al., 2015) and tulathromycin (Rutenberg et al., 2017; Venner et al., 2013a; Venner et al., 2013b), which are licensed for use in cattle, have been used, offering the advantage of reduced dosing frequency due to their prolonged half-lives compared with other macrolides (Berghaus et al., 2012; Venner et al., 2010). One study suggested tulathromycin’s potential ineffectiveness due to high minimum inhibitory concentrations (MICs) for *R. equi* (Carlson et al., 2010), whereas others did not reach definitive conclusions regarding its *in vivo* efficacy (Venner et al., 2013a; Venner et al., 2013b). Although no *in vitro* studies have assessed gamithromycin’s effect on *R. equi*, an *in vivo* study suggested its ability to limit disease progression (Hildebrand et al., 2015). These relatively recent suggestions, lacking robust scientific support, highlight the need to assess the equivalence among various macrolides and to determine their interchangeability in managing *R. equi* infections.

The frequent use of rifampicin in treatment raises concerns because it has recently been classified by the European Medicines Agency as a drug to avoid in veterinary medicine (European Medicines Agency, 2020). To promote more responsible use of antibiotics in animals, alternative options need to be considered—not only in anticipation of a potential prohibition of rifampicin in veterinary use but also because of the growing prevalence of *R. equi* isolates resistant to both macrolides and rifampicin (Giguère et al., 2017; Álvarez-Narváez et al., 2020; Álvarez-Narváez et al., 2021). Additionally, rifampicin is known to induce cytochrome P450 enzymes and drug transporters, thereby altering the pharmacokinetics of other drugs such as clarithromycin (Peters et al., 2011). When rifampicin and clarithromycin were co-administered to foals, the plasma concentration of clarithromycin was reduced by 90% (Peters et al., 2011; Peters et al., 2012). In this context, doxycycline has recently been proposed as an alternative to rifampicin (Wetzig et al., 2020), and its activity now requires validation.

To increase the predictive value of *in vitro* studies, the concentrations tested should closely replicate those achievable at the target site of action in foals following standard dosing regimens. The two primary sites of antimicrobial action against *R. equi* are the Pulmonary Epithelial Lining Fluid (PELF) and the intracellular compartments of macrophages, which are abundant in the lungs. Pharmacokinetic data for these sites, corresponding to doses routinely administered in horses, are available for most drugs used against *R. equi* (Table 1). Nevertheless, the majority of *in vitro* studies investigating antibiotic bactericidal activity against *R. equi* have used concentrations that do not accurately reflect these biophase levels (Giguère et al., 2012; Giguère et al., 2015). For instance, Giguère et al. (2012) reported tested concentrations of rifampicin and clarithromycin up to 64-fold the MIC of the strain, corresponding to levels 10 and 25 times lower than the maximum concentration achieved in foals, respectively. Conversely, the azithromycin and doxycycline concentrations tested were three times higher than the maximum concentrations achievable in foals. Another study by the same group tested concentrations found in foal plasma, which were 1.5–50 times lower than those reached intracellularly (Giguère et al., 2015), primarily because macrolides tend to accumulate within cells (Villarino and Martín‐Jiménez, 2013).

**TABLE 1:** Antibiotic concentrations (in µg/mL) tested based on the maximum concentration attained in in PELF and lung cells in foals.

	Tested concentrations (µg/mL)	
	Extracellular condition (RPMI pH 7.4 + 10% FBS)	Intracellular condition (MHB pH 5.8 + 10% FBS)
Rifampicin (Berlin et al., 2017)	10	5
Doxycycline (Womble et al., 2007)	10	10
Azithromycin (Giguère, 2017)	10	50
Clarithromycin (Jacks et al., 2002)	50	75
Gamithromycin (Berghaus et al., 2012)	5	10
Tulathromycin (Venner et al., 2010)	2	Not tested

Given that *R. equi* resides within macrophages, it can persist in various environments, such as cytosol, phagosomes, or phagolysosomes, which may have differing pH levels. Although it is well established that pH changes can influence antibiotic efficacy, particularly with macrolides (Dalhoff et al., 2005), no study has yet evaluated the impact of pH on antibiotic efficacy against *R. equi*.

This study was performed to assess the *in vitro* activity of six antimicrobial agents (including four macrolides) against *R. equi*, either alone or in combination, at concentrations and pH levels similar to those observed in the PELF and within macrophages in foals.

## 2 Materials and methods

### 2.1 Bacterial strain, cells, culture medium, and MIC determination

The virulent *R*. *equi* strain ATCC 33701 was used in this study. The presence of the *vapA* virulence gene was confirmed by PCR amplification as previously described (Anand et al., 2014). THP-1 cells (ATCC TIB-202) were cultured in RPMI supplemented with 10% decomplemented foetal bovine serum (FBS) in 75-cm^2^ plastic tissue culture flasks at 37°C with 5% CO_2_. Various bacterial culture media were prepared to simulate *in vivo* conditions. To replicate intracellular conditions at a pH of 5.8, Mueller–Hinton Broth (MHB) was supplemented with a buffer of mono- and di-basic sodium phosphates and 10% decomplemented FBS. To replicate extracellular conditions with a pH of 7.4 and to support the growth of THP-1 cells, RPMI with Glutamax and HEPES supplemented with 10% decomplemented FBS was used.

Among the antimicrobial agents, clarithromycin, tulathromycin, and gamithromycin were sourced from Glentham (Corsham, United Kingdom) and rifampicin, azithromycin, and doxycycline were sourced from Sigma (Saint-Quentin-Fallavier, France). Stock solutions were prepared in water for doxycycline, in DMSO for gamithromycin and tulathromycin, and in methanol for rifampicin, clarithromycin, and azithromycin.

The MIC for each antimicrobial agent (gamithromycin, clarithromycin, azithromycin, tulathromycin, rifampicin, and doxycycline) was determined using the standardized microdilution method provided by the Clinical Laboratory Standards Institute, with the exception of broth selection. Briefly, a bacterial inoculum of 5.10^5^ CFU/mL was prepared and exposed to different antibiotic concentrations achieved through serial half-dilutions in a 96-well plate. The MICs were assessed after 24 h of incubation at 37°C with 5% CO_2_. The MIC determinations were performed in triplicate for each medium.

### 2.2 Checkerboard assays

Checkerboard assays were conducted to determine the type of interaction between the antimicrobial drugs. Gamithromycin, clarithromycin and azithromycin were tested in combination with either rifampicin or doxycycline. In a 96-well plate, the first antibiotic underwent a vertical two-fold dilution, while the second antibiotic underwent a horizontal two-fold dilution. Following the same procedure as for MIC determination, a bacterial inoculum of 5.10^5^ CFU/mL was prepared and exposed to the antibiotics at 37°C with 5% CO_2_ for 24 h.

After 24 h of incubation, the Fractional Inhibitory Concentration (FIC) index was calculated as the sum of the FIC values for each drug in the combination. The FIC for each drug was determined by dividing the MIC of the drug when used in combination by the MIC of the same drug when used alone. An FIC index of ≤0.5 indicates synergy, an index between 0.5 and 4 suggests indifference, and an index of >4 indicates antagonism (Pillai et al., 2005). Each combination was tested in triplicate in each medium, and the final FIC index was calculated as the mean of the three replicates.

### 2.3 Time-kill studies

Time-kill studies (TKS) were conducted to evaluate the activity of each antibiotic, both individually and in combination, over time. The tested combinations were the same as those used in the checkerboard assay. The concentrations of antibiotics and the medium used in these TKS are provided in Table 1. To mimic extracellular conditions (RPMI), the maximum concentrations achievable in the PELF were used, while the highest intracellular concentrations attained in lung cells in foals were used to represent intracellular conditions (MHB).

A second set of experiments was carried out using clarithromycin at concentrations of 7.5, 2.25 and 0.5 μg/mL, corresponding to levels 10, 30, and 100 times lower than the maximum concentrations reported in Table 1. Doxycycline was tested at 5 and 1 μg/mL, corresponding to concentrations 2 and 10 times lower than the respective maximum concentrations.

Briefly, for each TKS, a bacterial inoculum of 2.5.10^5^ CFU/mL was prepared in the tested medium from an overnight culture. Bacteria were then exposed to each drug, either alone or in combination, at the specified concentrations for 72 h at 37°C with 5% CO_2_. At 0, 2, 6, 8, 24, 32, 48 and 72 h, 100 µL of the bacterial suspension was collected, centrifuged and washed with 0.9% sodium chloride. Serial 10-fold dilutions were then plated on Tryptic Soy Agar (TSA) plates supplemented with charcoal and magnesium sulphate and incubated overnight at 37°C. The limit of detection was 1.8 log_10_ CFU/mL. The activity of the drugs was considered bactericidal when the bacterial counts were reduced by more than 3 log_10_ CFU/mL. Each TKS was performed at least in triplicate for each condition.

### 2.4 Bactericidal activity after macrophage infection

THP-1 cells were transferred to a 25-cm^2^ flask at a density of 5.10^5^ cells/mL and then infected with *R. equi* to allow phagocytosis for 6 h at 37°C with 5% CO_2_, using a multiplicity of infection of 1. After the 6-hour phagocytosis period, doxycycline (10 or 1 μg/mL) and clarithromycin (50 or 5 μg/mL) were added and the cells were incubated for an additional 40 h. The negative control underwent a 46-hour incubation without *R. equi* or antibiotics.

Following this incubation period, the cells were washed three times with Phosphate-Buffered Saline and centrifuged at 300 *g*. The cell suspensions were then split into two aliquots for staining and bacterial counting. Fifty microlitres of the suspension were cytocentrifuged onto glass slides and stained using May–Grünwald–Giemsa (MGG). One millilitre of cell suspension was centrifuged again and resuspended in 1 mL of water to lyse the cells and release the bacteria. The bacteria were then counted as previously described.

### 2.5 Statistical analysis

Statistical analysis was conducted using GraphPad Prism software version 8.4.2. Analysis of variance was used to compare the effects of the antibiotics, with statistical significance set at *p* < 0.05.

## 3 Results

### 3.1 MIC determinations

The MICs of rifampicin, doxycycline and four macrolides (clarithromycin, azithromycin, gamithromycin, and tulathromycin) for the *R. equi* ATCC 33701 strain in different media are presented in Table 2. Various media were tested to evaluate the influence of FBS and pH on the MIC values. Among the four macrolides, clarithromycin exhibited significantly lower MICs than azithromycin, gamithromycin and tulathromycin across all five media. The presence of FBS slightly influenced the MICs of azithromycin and gamithromycin. At pH 7.4, tulathromycin had MIC values 32 to 500 times higher than those of the other macrolides. The antimicrobial activity of the four tested macrolides was also affected by pH, with the MICs differing by a factor of 4–128 between pH 5.8 and 7.4. For doxycycline, the MIC varied by a factor of 4 between pH 5.8 and 7.4. Notably, the MICs of rifampicin remained consistently low and were unaffected by changes in pH.

**TABLE 2:** MIC (in µg/mL) of rifampicin, doxycycline, clarithromycin, azithromycin, gamithromycin, and tulathromycin against *Rhodococcus equi* ATCC 33701 strain in standard broth (MHB), MHB with 10% FBS, MHB with 10% FBS at pH 5.8 and RPMI at pH 7.4 and 5.8.

	MIC (µg/mL)				
	MHB	MHB + 10% FBS	MHB + 10% FBS pH 5.8	RPMI + 10% FBS pH 7.4	RPMI + 10% FBS pH 5.8
Rifampicin	0.064	0.032	0.064	0.064	0.064
Doxycycline	0.25	0.25	0.25	1	0.25
Clarithromycin	0.064	0.032	0.5	0.032	0.5
Azithromycin	2	0.5	64	0.5	64
Gamithromycin	2	0.5	64	0.5	64
Tulathromycin	64	32	64	16	64

### 3.2 Type of interactions between drugs in combinations

The interactions between macrolides and either rifampicin or doxycycline on the *R. equi* ATCC 33701 strain were evaluated using checkerboard assays. The FIC indices for each pairwise comparison are presented in Table 3. All tested combinations appeared to be indifferent because the FIC index ranged from 0.5 to 4.0 at both pH 5.8 and 7.4, with the exception of the combination of doxycycline and azithromycin, which had a FIC index slightly below 0.5 at pH 5.8.

**TABLE 3:** FIC index for the combination of rifampicin or doxycycline with macrolides for *Rhodococcus equi* ATCC 33701 strain at pH 5.8 and 7.4.

	FIC index	
	MHB + 10% FBS pH 5.8	RPMI + 10% FBS pH 7.4
Rifampicin/clarithromycin	1	0.75
Rifampicin/azithromycin	1	1
Rifampicin/gamithromycin	1	0.75
Doxycycline/clarithromycin	0.59	0.64
Doxycycline/azithromycin	0.45	0.52
Doxycycline/gamithromycin	0.64	0.50

### 3.3 Bactericidal activity of antibiotics alone or combined

**FIGURE 1:**
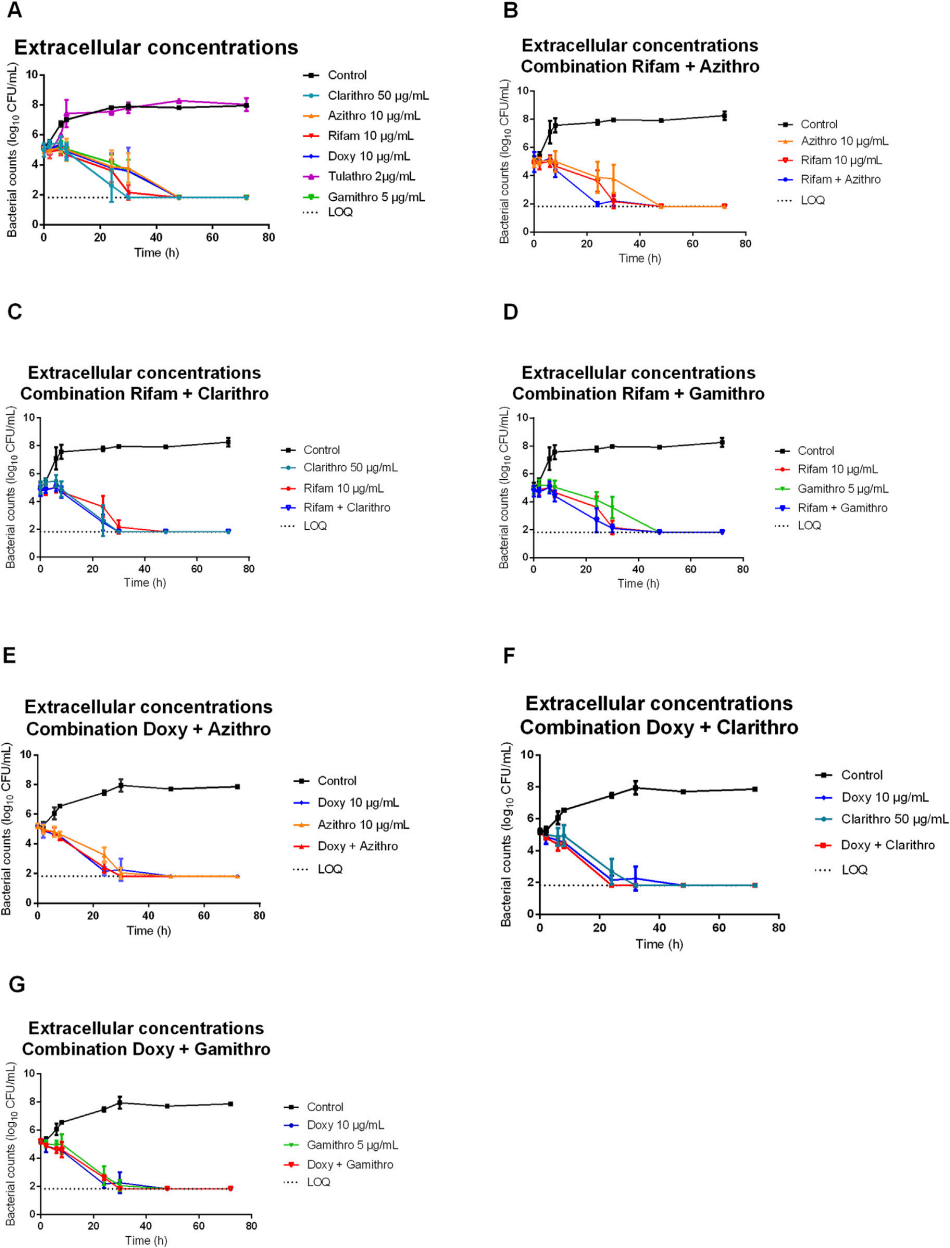
Bacterial counts of *Rhodococcus equi* strain ATCC 33701 under extracellular conditions (RPMI + 10% FBS at pH 7.4) following exposure to macrolides [clarithromycin (clarithro) (50 μg/mL), gamithromycin (gamithro) (5 μg/mL), azithromycin (azithro) (10 μg/mL), tulathromycin (tulathro) (2 μg/mL)], rifampicin (rifam) (10 μg/mL) or doxycycline (doxy) (10 μg/mL) **(A)** alone and **(B–G)** in combination: **(B)** azithromycin/rifampicin, **(C)** clarithromycin/rifampicin, **(D)** gamithromycin/rifampicin, **(E)** azithromycin/doxycycline, **(F)** clarithromycin/doxycycline, **(G)** gamithromycin/doxycycline. Each point on the curve represents the mean value of three independent experiments. Tulathromycin is significantly different from the other antibiotics (*p* < 0.05).

**FIGURE 2:**
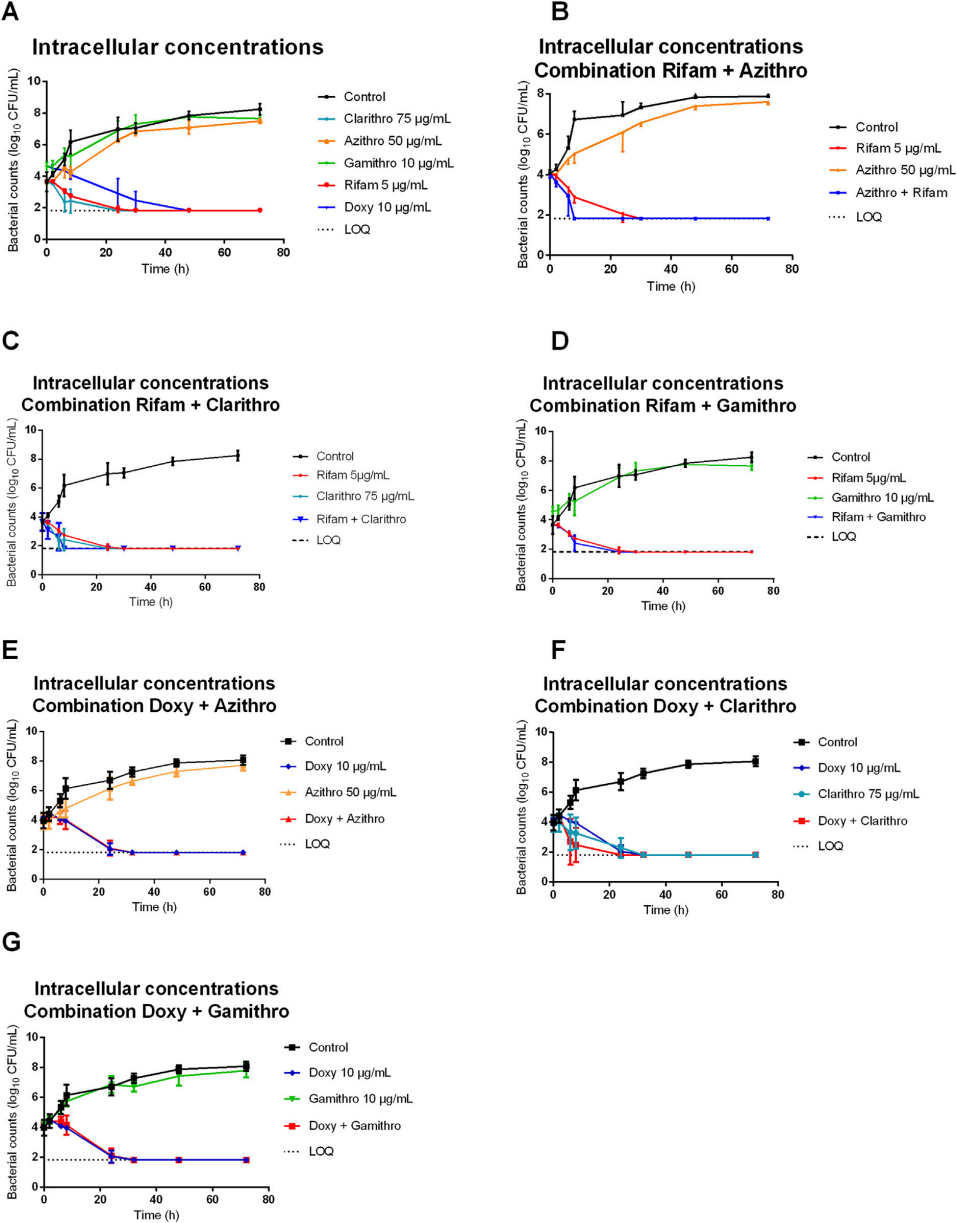
Bacterial counts of *Rhodococcus equi* strain ATCC 33701 under intracellular conditions (MHB +10% FBS at pH 5.8) following exposure to macrolides [clarithromycin (clarithro) (50 μg/mL), gamithromycin (gamithro) (5 μg/mL), azithromycin (azithro) (10 μg/mL)], rifampicin (rifam) (10 μg/mL) or doxycycline (doxy) (10 μg/mL) **(A)** alone and **(B–G)** in combination **(B)** azithromycin/rifampicin, **(C)** clarithromycin/rifampicin, **(D)** gamithromycin/rifampicin, **(E)** azithromycin/doxycycline, **(F)** clarithromycin/doxycycline, **(G)** gamithromycin/doxycycline. Each point on the curve represents the mean value of three independent experiments.

The antimicrobial activity of the macrolides (clarithromycin, azithromycin, gamithromycin and tulathromycin), rifampicin and doxycycline, both individually and in combination at the concentrations specified in Table 1, was evaluated against the *R. equi* ATCC 33701 strain using TKS over a 72-hour period. The results of the TKS conducted under extracellular and intracellular conditions (different media and pH) are shown in Figures 1, 2, respectively.

Under extracellular conditions (RPMI pH 7.4 + 10% FBS), rifampicin, clarithromycin, gamithromycin, azithromycin and doxycycline alone demonstrated significant efficacy, effectively reducing the bacterial count below the detection limit within 24–48 h. By contrast, tulathromycin’s effect on bacterial counts closely mirrored that of the positive control (Figure 1A). As a result, tulathromycin was not further tested in this study.

Under intracellular conditions (MHB pH 5.8 + 10% FBS), gamithromycin and azithromycin showed no bactericidal effect, with bacterial counts reaching a plateau of approximately 8 log_10_ CFU/mL up to 72 h, similar to the positive control (Figure 2A). Notably, clarithromycin, doxycycline and rifampicin exhibited a reduction in the bacterial count below the detection limit between 24 and 32 h, similar to their activity observed under extracellular conditions (Figure 1A).

When examining TKS with combinations of antibiotics, similar results were observed under both extracellular and intracellular conditions (Figures 1B–G, 2B–G). Combining azithromycin with rifampicin enhanced the rate of bacterial killing compared with the individual drugs, resulting in bacterial eradication between 8 and 24 h (Figures 1B, 2B). The increased killing rate was less pronounced for the combination of gamithromycin and rifampicin (Figures 1D, 2D). For clarithromycin, which already had the fastest killing activity when used alone, the addition of rifampicin did not further enhance its bactericidal effect (Figures 1C, 2C).

Combining azithromycin or gamithromycin with doxycycline did not result in increased bacterial killing compared with doxycycline alone (Figures 1E–G, 2E–G). However, the addition of clarithromycin to doxycycline appeared to slightly enhance the bactericidal activity compared with doxycycline alone (Figures 1F, 2F).

To identify alternatives to rifampicin, we further investigated the activity of doxycycline and clarithromycin by assessing their effects at lower concentration ranges.

**FIGURE 3:**
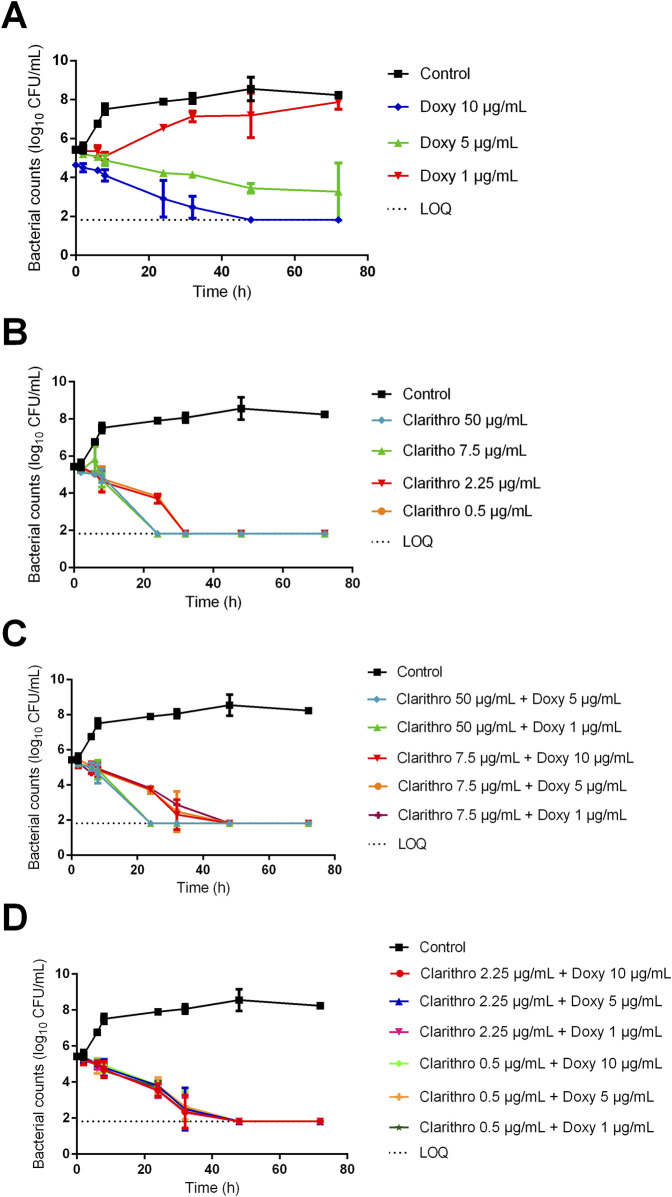
Bacterial counts of *Rhodococcus equi* strain ATCC 33701 under extracellular conditions (RPMI + 10% FBS at pH 7.4) following exposure to **(A)** doxycycline (10 μg/mL, 5 μg/mL, 1 μg/mL), **(B)** clarithromycin (clarithro) (50 μg/mL, 7.5 μg/mL, 2.25 μg/mL, 0.5 μg/mL), and **(C, D)** the drugs in combination. Each point on the curve represents the mean value of three independent experiments.

Under extracellular conditions (RPMI pH 7.4 + 10% FBS), doxycycline at 5 μg/mL reduced bacterial counts by 2 log_10_ CFU/mL at 72 h, with no bactericidal effect observed at 1 μg/mL (Figure 3A). The effects of these concentrations were significantly different (*p* < 0.05). Clarithromycin at 7.5 μg/mL demonstrated comparable efficacy to that at 50 μg/mL, reducing bacterial counts below the detection limit within 24 h. Clarithromycin at 2.25 and 0.5 μg/mL showed a similar trend, with bacterial elimination slightly delayed to 32 h (Figure 3B). However, combining doxycycline and clarithromycin at any concentration did not result in a faster bactericidal effect compared to clarithromycin alone (Figures 3C, D).

**FIGURE 4:**
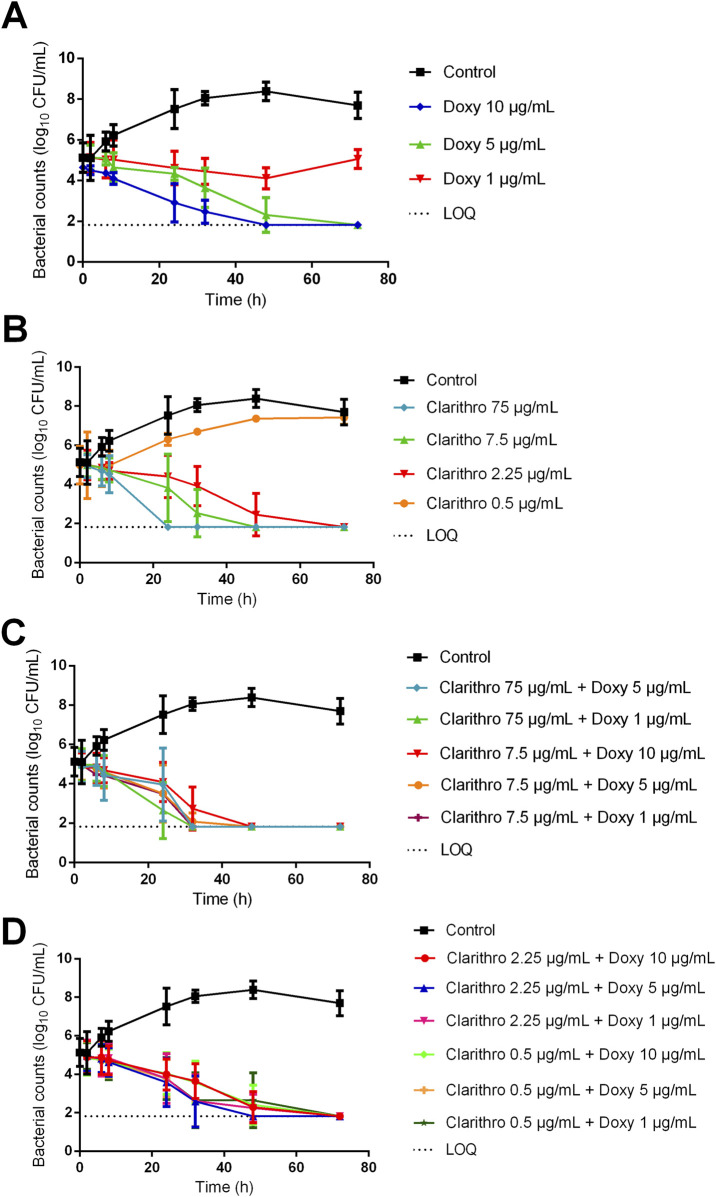
Bacterial counts of *Rhodococcus equi* strain ATCC 33701 under intracellular conditions (MHB + 10% FBS at pH 5.8) following exposure to **(A)** doxycycline (10 μg/mL, 5 μg/mL, 1 μg/mL), **(B)** clarithromycin (clarithro) (50 μg/mL, 7.5 μg/mL, 2.25 μg/mL, 0.5 μg/mL), and **(C, D)** the drugs in combination. Each point on the curve represents the mean value of three independent experiments.

Under intracellular conditions (MHB pH 5.8 + 10% FBS), doxycycline exhibited greater activity than in extracellular conditions. The effects obtained with 5 and 1 μg/mL were significantly different. Doxycycline at 5 μg/mL reduced bacterial counts below the detection limit at 72 h, whereas 1 μg/mL led to a slight reduction of less than one log_10_ CFU/mL at 48 h, followed by regrowth (Figure 4A). Clarithromycin displayed a slower decrease in bacterial counts compared with extracellular conditions. Clarithromycin concentrations of 7.5 and 2.25 μg/mL led to counts below the detection limit only at 48 h and 72 h, respectively, while 0.5 μg/mL had a significantly lower effect, showing no killing effect on bacteria, reaching the positive control curve at 72 h (Figure 4B). Similar to extracellular conditions, combinations of doxycycline and clarithromycin did not result in faster killing than clarithromycin alone, except for the combination of the lowest concentrations of clarithromycin (0.5 μg/mL) and doxycycline (1 μg/mL). This combination resulted in bacterial counts below the detection limit at 72 h, whereas each antibiotic alone had no killing effect at this time point (Figures 4C, D).

### 3.4 Bactericidal activity of doxycycline and clarithromycin on phagocytized *Rhodococcus equi*

**FIGURE 5:**
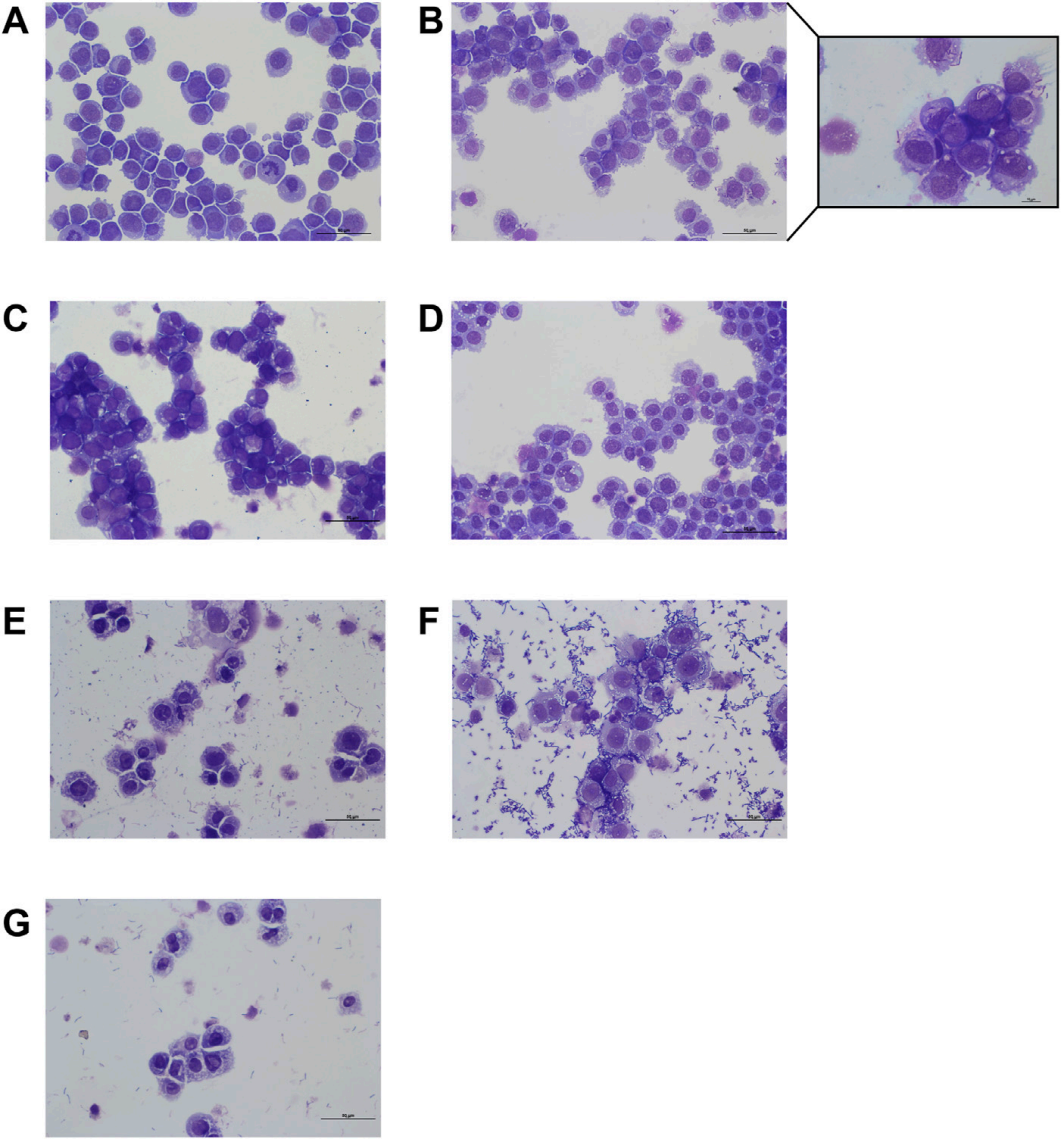
Microscopic examination of THP-1 macrophages (ATCC TIB-202) infected with *Rhodococcus equi* strain ATCC 33701 and exposed to antibiotics for 40 h. Suspensions of cells were centrifuged onto glass slides and stained with MGG. **(A)** Negative control. **(B)** Phagocytosis for 6 h followed by 40 h of treatment with doxycycline (10 μg/mL). **(C)** Clarithromycin (50 μg/mL). **(D)** Doxycycline (10 μg/mL)/clarithromycin (50 μg/mL). **(E)** Clarithromycin (0.5 μg/mL). **(F)** Doxycycline (1 μg/mL). **(G)** Doxycycline (1 μg/mL)/clarithromycin (0.5 μg/mL). Scale = 50 μm and 10 µm.

**FIGURE 6:**
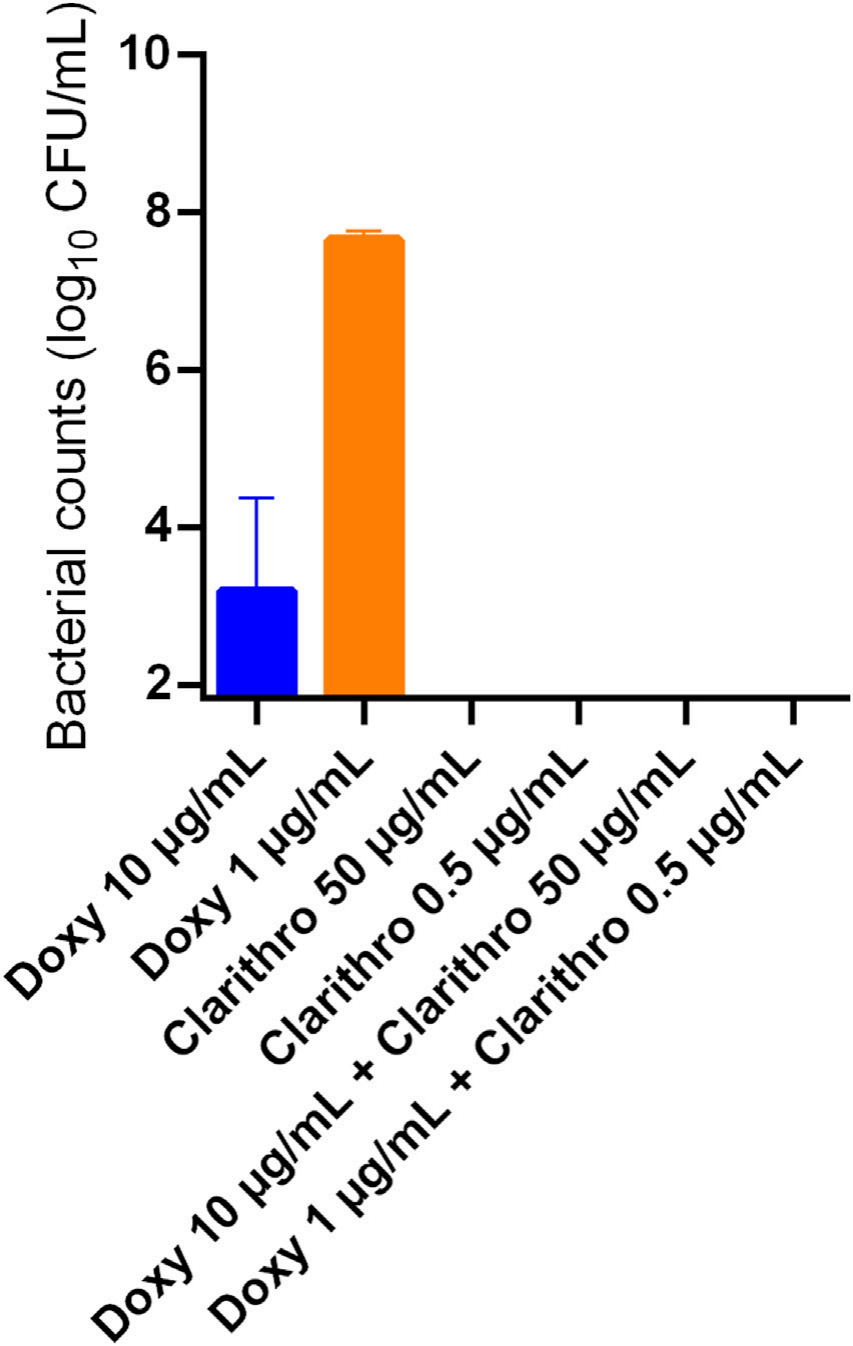
Bacterial counts of *Rhodococcus equi* strain ATCC 33701 after phagocytosis by THP-1 macrophages (ATCC TIB-202) and exposure to clarithromycin (clarithro) (50 μg/mL, 0.5 μg/mL), doxycycline (doxy) (10 μg/mL, 1 μg/mL) and combinations of doxycycline (10 μg/mL)/clarithromycin (50 μg/mL) or doxycycline (1 μg/mL)/clarithromycin (0.5 μg/mL) for 40 h. Bacteria were counted after three washes of cell suspensions followed by cell lysis. Each value represents the mean of three independent experiments.

The efficacy of clarithromycin and doxycycline against both extracellular and intracellular *R. equi* infection was evaluated using macrophage infection assays. *Rhodococcus equi* was phagocytized for 6 h and then exposed to antibiotics for 40 h. Depending on the drugs and concentrations tested, varying quantities of bacteria were observed on the slides. Clarithromycin at 50 μg/mL demonstrated *in vitro* activity, with no bacteria detected in THP-1 cells (Figures 5C, 6), similar to the negative control without bacteria (Figure 5A). With doxycycline at 10 μg/mL, a few bacteria were still visible in the medium and within the vacuoles of cells (Figure 5B), with bacterial counts of 8.5.10^3^ CFU/mL after cell lysis (Figure 6). When these two antibiotics were combined, complete elimination of *R. equi* was achieved as shown by the fact that no bacteria were observed or counted (Figures 5D, 6).

In follow-up assessments at lower concentrations, clarithromycin at 0.5 μg/mL and doxycycline at 1 μg/mL showed reduced effectiveness against *R. equi*, with more bacteria observed both inside and outside the cells compared with the conditions with 50 μg/mL clarithromycin or 10 μg/mL doxycycline (Figures 5E, F). Bacterial counts after cell lysis confirmed the presence of 4.67.10^7^ CFU/mL following exposure to doxycycline at 1 μg/mL, whereas bacterial counts were below the limit of quantification after exposure to clarithromycin at 0.5 μg/mL (Figure 6). After exposure to the combination of clarithromycin at 0.5 μg/mL and doxycycline at 1 μg/mL, very few bacteria were visible on the slide (Figure 5G), likely too few or too fragile to be counted on plates (Figure 6).

## 4 Discussion

*Rhodococcus equi* infections are prevalent worldwide and pose a significant financial burden to equine breeders. The current standard treatment involves a combination of rifampicin with a macrolide, either clarithromycin or azithromycin. In parallel to the use of rifampicin, an antibiotic of critical importance for human health [Antimicrobial Advice *ad hoc* Expert Group (AMEG) Category A (European Medicines Agency, 2020)], that is being questioned in veterinary medicine, the availability of other macrolides (such as tulathromycin and gamithromycin) necessitates a fresh perspective to guide the selection of optimal drugs.

The goals of the present study were to evaluate the activity of antibiotics against *R. equi* by exposing the bacteria to concentrations similar to those found at the target sites in foals. Additionally, there is a need for a better understanding of the effect of pH on antibiotic activity against *R. equi*, given its ability to survive and multiply intracellularly. *Rhodococcus equi* experiences different pH conditions, transitioning from the phagosome, where the pH decreases, to the phagolysosome, which is characterised by a significantly lower pH following fusion with a lysosome (Sivaloganathan and Brynildsen, 2021).

For the antibiotics tested, the MIC results obtained at pH 7.4 in this study align with previously reported MICs for the same strain (Giguère et al., 2015; Carlson et al., 2010) and other strains (Giguère et al., 2012; Prescott and Nicholson, 1984; Berghaus et al., 2013; Erol et al., 2022). The MICs of these antibiotics at pH 5.8 confirmed that a lower pH leads to an increase in the MIC for macrolides (Dalhoff et al., 2005; Retsema et al., 1991; Hardy et al., 1988). The MIC values of azithromycin, gamithromycin and tulathromycin in an acidic environment were even higher than the maximum concentrations found in lung cells, which could potentially explain therapeutic failures. A previous study using TKS and checkerboard assays demonstrated slight synergy between rifampicin and clarithromycin and significant synergy between doxycycline and clarithromycin, as well as between doxycycline and azithromycin (Giguère et al., 2012). By contrast, another study involving different strains showed indifference between rifampicin and clarithromycin and weak synergy between doxycycline and clarithromycin, indicating that antibiotic activity is highly strain-dependent (Erol et al., 2022). In the present study, the checkerboard assay did not show any synergy between these antibiotics, except for the combination of doxycycline and azithromycin. However, our study used different media and strains, which could account for these differences. Nonetheless, no antagonism was observed, suggesting that at least from a pharmacodynamic perspective, any of the tested combinations could potentially be used in foals.

One limitation of assessing interactions through the checkerboard method is its reliance on observing turbidity, which means only concentrations close to the MIC can be investigated. The antibiotics tested in this study reach concentrations in foals that can differ significantly from the MIC values. As a result, checkerboard assays have limitations in providing general information on the type and intensity of interactions that might occur *in vivo*. Therefore, in this study, time-kill curves were performed over 72 h, using the maximum concentrations achieved in PELF at pH 7.4 or in alveolar cells of the foal at acidic pH to replicate the conditions encountered in the animal. Time-kill curves for the macrolides indicated a lack of effect of tulathromycin, while clarithromycin enabled bacterial elimination within 24 h. Gamithromycin and azithromycin showed efficacy only under extracellular conditions, with no effect on *R. equi* at pH 5.8, consistent with their very high MICs at this pH. This finding underscores the non-equivalence of macrolides. It is important to note that uncertainties exist regarding the intracellular concentrations of drugs because they are highly dependent on cellular compartments and their respective pH levels. Additionally, *R. equi* has been suggested to hinder acidification of the phagolysosome (Von Bargen et al., 2009), preventing it from reaching as low as pH 5.8. Therefore, our study may have underestimated the activity of macrolides under intracellular conditions. Indeed, despite the absence of activity of gamithromycin in intracellular conditions *in vitro*, Hildebrand et al. (2015) demonstrated that gamithromycin could prevent disease progression *in vivo*. Even when focusing solely on extracellular conditions, which are less subject to debate, our results still demonstrate that macrolides have varying activities and cannot be used interchangeably in the context of *R. equi* infection.

Time-kill curves for macrolide and rifampicin combinations indicated that combining rifampicin with azithromycin slightly increased the bacterial killing rate compared with each antibiotic alone. The combination of rifampicin with clarithromycin, the macrolide with the most rapid killing activity on its own, did not enhance bactericidal activity under any conditions compared with clarithromycin alone. Furthermore, it has been demonstrated that combining rifampicin with clarithromycin causes a significant reduction in plasma concentrations of clarithromycin in foals (Peters et al., 2012), which could compromise both its intracellular and extracellular activity *in vivo*. Doxycycline, which does not affect the pharmacokinetics of clarithromycin and is recommended as a first-line antibiotic [AMEG Category D (European Medicines Agency, 2020)], was tested as an alternative to rifampicin. Doxycycline is suitable for use in horses (Dunkel and Johns, 2015) and has very few adverse effects (Womble et al., 2007; Perez et al., 2022). Pharmacokinetic data for horses are also readily available (Womble et al., 2007). Additionally, studies have shown that *R. equi* strains resistant to rifampicin and clarithromycin remain susceptible to doxycycline (Álvarez-Narváez et al., 2021), and the occurrence of doxycycline resistance is rare, affecting less than 1% of isolates (Giguère et al., 2017; Berghaus et al., 2015). In this study, at maximal achievable concentrations in foals, the combination of doxycycline with macrolides showed either no enhancement (with azithromycin and gamithromycin) or only a slight enhancement (with clarithromycin) in the bacterial killing rate compared with the drugs used alone.

After a first set of experiments using maximal local concentrations reported in the literature, we further explored the combination of doxycycline and clarithromycin as a potential substitute for rifampicin-containing combinations by examining their activity at lower concentrations. Given the half-life of approximately 8 h for doxycycline (Womble et al., 2007) and 4 h for clarithromycin (Jacks et al., 2002) in horses, the antibiotic levels in the PELF and intracellularly would be approximately 10 and 100 times lower than the maximum concentrations for doxycycline and clarithromycin, respectively, 24 h post-administration. At these lower concentrations, which are likely the minimum levels during treatment, the drugs alone demonstrated no efficacy under intracellular conditions. However, when combined, they exhibited bactericidal activity, suggesting that the combination could maintain effectiveness throughout the treatment period. This synergy may also offer flexibility in spacing out antibiotic administrations.

A limitation of this study is that phagocytosis assays were performed using undifferentiated THP-1 macrophages, which may reduce the cells’ phagocytic efficiency (Shiratsuchi and Basson, 2004). Nonetheless, bacteria were visualized within vacuoles by microscopy, confirming phagocytosis and allowing assessment of the intracellular activity of clarithromycin and doxycycline. This experiment showed that clarithromycin’s effectiveness in cells might be slightly higher than doxycycline’s and confirmed that the combination of both drugs could lead to bacterial eradication even at low concentrations.

In conclusion, clarithromycin demonstrated high *in vitro* activity against *R. equi*, and its effectiveness has been confirmed in an *in vivo* study (Giguére et al., 2004). The use of tulathromycin should be discouraged because of its significantly lower *in vitro* activity compared with other macrolides, rifampicin and doxycycline. The necessity of a second drug alongside clarithromycin is debatable, considering its excellent standalone activity. However, if a combination is deemed desirable for treating *R. equi* infections, the combination of doxycycline and clarithromycin appears promising as a substitute for rifampicin. Nevertheless, further studies are needed to evaluate the clinical efficacy and potential side effects of doxycycline in foals.

## Data Availability

The raw data supporting the conclusions of this article will be made available by the authors, without undue reservation.
